# Does Growth Stunting Correlate with Oral Health in Children?: A Systematic Review

**DOI:** 10.1055/s-0041-1731887

**Published:** 2021-10-01

**Authors:** Zayyana Jasmine Sadida, Ratna Indriyanti, Arlette Suzy Setiawan

**Affiliations:** 1 Faculty of Dentistry, Universitas Padjadjaran, Jl. Sekeloa Selatan 1, Bandung, Indonesia; 2 Department of Pediatric Dentistry, Faculty of Dentistry, Universitas Padjadjaran, Jl. Sekeloa Selatan 1, Bandung, Indonesia

**Keywords:** growth stunting, oral hygiene, salivary flow rate, plaque, malnutrition, dental caries

## Abstract

Growth stunting is when children tend to be shorter than their peers through the World Health Organization child growth standard measurement. The condition may affect the development of the brain and other parts of the body, including the oral cavity, which manifests in oral hygiene and overall oral health. This systematic approach literature study aimed to evaluate the correlation between growth stunting and oral health in children. This study was conducted by using a literature review method with a systematic approach by searching for articles related to research topics on PubMed and Google Scholar. The search was adjusted to the inclusion category, which is research that discusses malnutrition and oral health published between 2010 and 2020—research conducted on boys and girls—from birth to 18 years. The exclusion categories used were articles that did not discuss growth stunting and oral health, and grey literature was excluded. The selection of articles was carried out by using the Preferred Reporting Items for Systematic Reviews and Meta-Analyses (PRISMA) approach and resulted in 10 selected articles with details as follows: the correlation between growth stunting and oral health in three articles. A high plaque index in growth stunting children was found in two articles, and a decrease in saliva composition in growth stunting children was also found in two articles. Four articles showed a relationship between growth stunting and salivary flow rate. Lastly, one article showed the relationship between growth stunting and the incidence of dental caries. Despite the limitation of the review, the correlation between growth stunting and overall oral health in children can be seen through the oral hygiene index as moderate to low, high plaque index, decreased salivary flow rate, salivary composition, and dental caries in children with growth stunting.

## Introduction

Growth stunting is a form of growth failure in the body and brain in children caused by long-term malnutrition or recurrent infections so that children tend to be shorter than their peers and have delays in thinking processes. 1 Stunting can be measured based on the World Health Organization (WHO) child growth standards median standard deviation with body height measurement to age (TB/U) indicator, namely if the z-score value is TB/U < −2 SD. 2 A total of approximately 21.9% or 149 million children under 5 years of age in the world are stunted, with 25% found in Southeast Asia. 3 Indonesian Basic Health Research in 2018 states that the prevalence of growth stunting in Indonesia is 30.8%. This prevalence indicates a decrease of 6.4% compared to the prevalence in 2013 (37.2%). 4

Clinical manifestations of growth stunting generally affect motoric and mental development. 5 Whereas in the oral cavity, stunting manifests as a lack of resistance to microbial biofilms and decreased salivary flow rate. 6 7 This manifestation can affect a person’s oral health, which has a vital role in the quality of life of a child and provides an overview of general health until the child grows up. 8 9 Poor oral hygiene will lead to dental and oral health problems. 8 Based on Basic Health Research Data in 2018, the proportion of dental and oral health problems in Indonesia is 57.6%, which means it is still relatively high. 10

Caries and other oral diseases may develop from plaque deposits that resulted from the inability of a child to brush their teeth due to inadequate psychomotor development. Problems in psychomotor development are often seen as the later effects of growth stunting. 11 The formation and adhesion of plaque biofilm can be prevented by the components in saliva, causing a salivary flow to have an essential role in preventing the adherence of bacteria to the teeth and oral mucosal surfaces. 12 Singh et al states a decrease in salivary flow rate in chronically malnourished children (growth stunting), both stimulated and unstimulated. 13 This inhibition of salivary flow is caused by atrophy of the salivary glands associated with protein deficiency (protein energy malnutrition) and vitamin A, thereby reducing the cavity defense capacity against infection buffering ability against plaque acid. This condition may also affect the amount and composition of Saliva, limiting the protective effect in the oral cavity, thereby affecting one’s oral health. This review evaluates a correlation between growth stunting and oral health in children with growth stunting experience.

## Methods

### Primary Outcome

The primary outcome of this systemic review is to evaluate the correlation between growth stunting and oral health in children aged up to 5 years old with a history of stunting.

### Inclusion/Exclusion Criteria

The eligibility criteria were defined by a selection of articles in inclusion and exclusion criteria. The articles were identified as already in the correct population, using appropriate interventions, and relevant to the research topic. 14 After all relevant articles were submitted and duplicated removed, titles and abstracts from all remaining articles were screened manually to remove articles that did not meet the inclusion criteria. References from included articles needed to be checked to identify other relevant studies that could be re-entered based on predefined inclusion criteria. 15

Articles that report a study that did not examine the association of stunting with oral health in children were excluded. Materials and research produced by an organization outside of the traditional commercial or academic publishing and distribution channels were also excluded; these articles were known as grey literature. Common grey literature publication types include reports (annual, research, technical, project), working papers, or government documents. 16

### Search Strategy and Study Selection

A comprehensive search of the PubMed and Google Scholar databases from its inception to December 2020 was conducted to identify studies that evaluated the main topic of this systematic review. The search was carried out by using keywords or MESH terms and the article text for the following search terms: (“stunting”), (“chronic malnutrition”), (“malnutrition”), and (“oral health).” These keywords were then used together with the Boolean operators (OR and AND) to combine the search. The filters used in PubMed were the publication year filter, which is 2010 to 2020, and the age filter for children (0–18 years), while the filters used in Google Scholar were the year of publication filter within the same period.

The articles resulting from this search were screened manually—first based on the title, then the abstract, and finally, the complete manuscript—to determine their appropriateness for inclusion in the literature review. References cited in the included articles were also reviewed to identify additional published articles not identified by the database search.

### Data Extraction

Selected publications were independently reviewed by two investigators (Z.J. and R.A.). The extracted data included information about the study design characteristics, group, and subjects characteristics. The data also include the author’s name, country, year of publication, keywords, research method, sample, assessment, research results, conclusions, and the quality level of each article. Disagreements between the authors were resolved through consensus.

Quality assessment of all included articles was performed independently by a reviewer as part of the data extraction process. Quality assessment or critical appraisal is a systematic assessment and interpretation of research results to consider the validity, results, and relevance of research. 17 The quality assessment used in this study is the National Institutes of Health (NIH) quality assessment tool. 18 Reviewers used the study rating tools on the range of items included in each tool to judge each study to be of “good,” “fair,” or “poor” quality. The ratings on the different items were used by the reviewers to assess the risk of bias in the study due to flaws in study design or implementation. In general terms, a “good” study has the least risk of bias, and results are considered to be valid. A “fair” study is susceptible to some bias deemed not sufficient to invalidate its results. The fair quality category is likely to be broad, so studies with this rating will vary in their strengths and weaknesses. A “poor” rating indicates significant risk of bias. Studies rated poor were excluded from the body of evidence to be considered for each check question (CQ). The only exception allowed was if there was no other evidence available, then poor quality studies could be considered. However, this exception was not applied in this project because there were no situations found where only poor quality studies were available for a body of evidence for a particular CQ. 19

The quality of the reported information included in each article was assessed by following the Preferred Reporting Items for Systematic Review and Meta-analyses (PRISMA).

## Results

### Studies Included

**Fig. 1:**
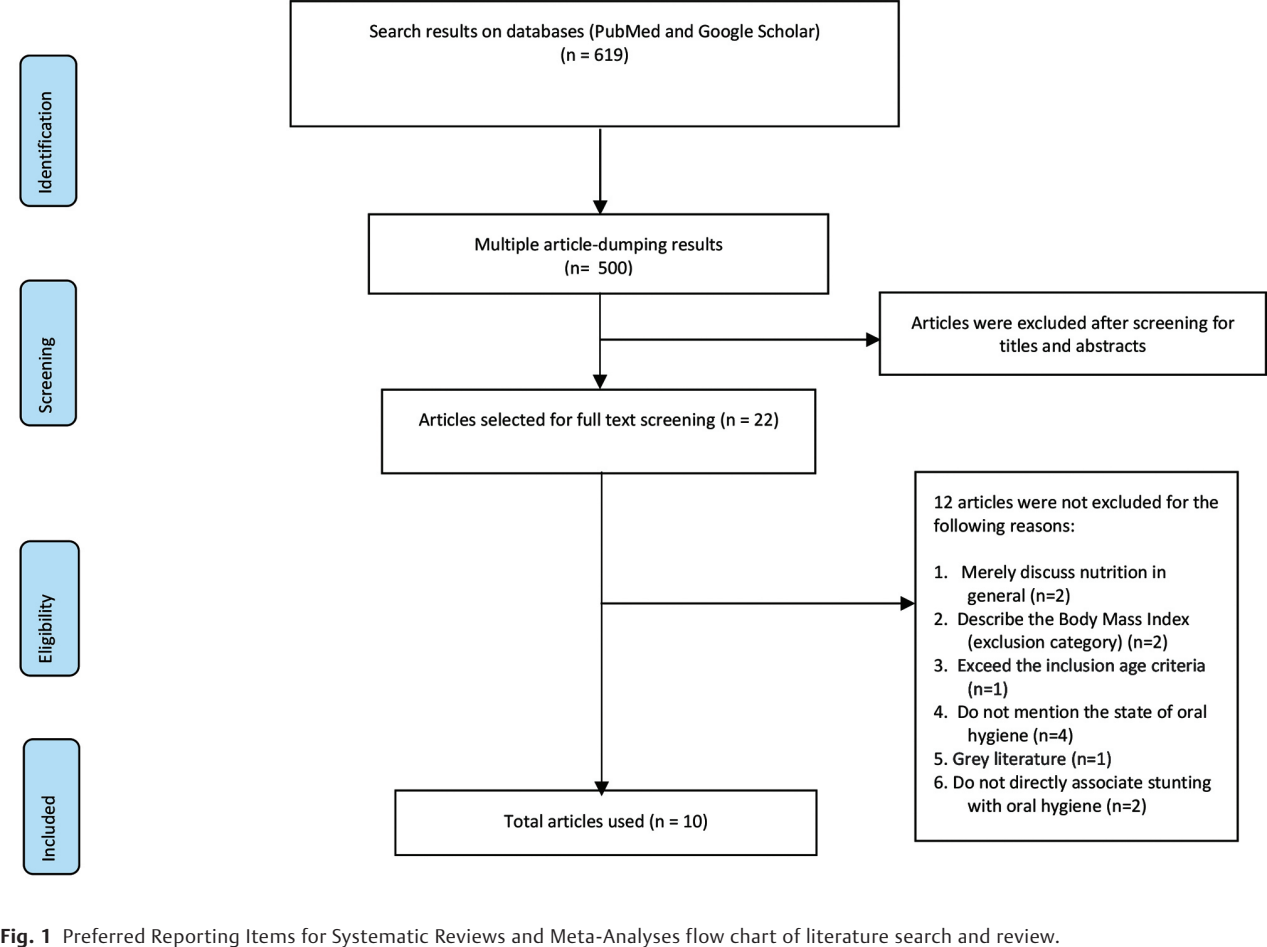
Preferred Reporting Items for Systematic Reviews and Meta-Analyses flow chart of literature search and review.

The study selection process for inclusion in the review is summarized in Fig. 1 . The database search strategy identified 619 potentially eligible references, which resulted in 500 after multiple article dumping. After screening titles and abstracts, 22 full-text articles were reviewed in their entirety. Twelve articles were excluded because of the following reasons; merely discuss nutrition in general ( *n* = 2), describe the body mass index (exclusion category; *n* = 2), exceed the inclusion age criteria, do not mention the state of oral health ( *n* = 4), grey literature, and do not directly associate growth stunting with oral health ( *n* = 2). Eventually, 10 articles were included in the literature research ( Table 1 ).

**Table 1:** Results of literature research

No.	Study (Year)	Journal title	Research method	*n*	Assessment	Results	Conclusion	Biased examination
1	Folayan et al (2020) 34	Association between early childhood caries and malnutrition in a suburban population in Nigeria	Cross-sectional	370 (6 mo–12 y)	OHI-S	Factors associated with ECC are stunting, underweight, overweight, and moderate oral hygiene. ECC prevalence is 66% lower in stunted children (APR: 0.14; 95% CI: 0.03–0.69; *p* = 0.02) and almost seven times higher in overweight children (APR: 6.88; 95% CI: 1.83– 25.85; *p* < 0.001) compared to normal children. In children with ECC, oral cavity hygiene is considered moderate, and none has poor oral hygiene (APR: 0; 95% CI: 0–0; *p* < 0.000).	There is a relation between ECC and nutrition status in children. Children with ECC have moderate oral hygiene. This research does not find any relation between ECC with consumption of sweets, so moderate oral hygiene is related to malnutrition in children.	Good
2	Vieira et al (2020) 22	Chronic malnutrition and oral health status children aged 1 to 5 years	Cross-sectional	82 (12–71 mo)	Salivary flow	69 out of 82 observed children show low to insufficient buffer capacity. A significant difference in salivary flow is found in various nutrition status categories. (ANOVA, *p* < 0.05). Coefficient correlation shows the negative correlation between nutrition status with the salivary flow ( *r* = −0.267).	Salivary flow decreases along with the increase of malnutrition, enabling it to worsen children’s vulnerability to dental caries and opportunistic infection. There is a correlation between malnutrition and the level of salivary flow.	Good
3	Achmad et al (2020) 28	Analysis of dental caries and gingivitis with the occurrence of stunting in children in Makassar City	Cross-sectional	208	def-t	There is a correlation between teeth condition and the incidence of stunting in children.	There is a correlation between stunting in children with children’s oral health as seen in the result of the study which stated that a significance difference at the level of caries in stunting children.	Poor
4	Vargas-Palomino et al (2019) 24	Oral health and oral hygiene conditions and nutritional status in children attending a health facility in The Huanuco Region, Peru	Cross-sectional	118 (3–5 y)	OHI-S	In 25 stunted children, 24 have poor oral hygiene and 1 with moderate oral hygiene. Research results prove to be significant statistically with ( *p* < 0.001).	Malnutrition affects children's oral health and hygiene.	Poor
5	Singh et al (2018) 21	Association of nutritional status on salivary flow rate, dental caries status and eruption pattern in pediatric population in India	Cross-sectional	97 (5–12 y)	Salivary flow	The unstimulated SFR score is 0.53 ± 0.15 mL/min (Group I), 0.14 ± 0.04 mL/minute (Group II), 0.21 ± 0.20 mL/min (Group III) proven significant statistically with ( *p* = 0.001). For stimulated SFR, the score is 1.94 ± 0.44 mL/min (Group I), 1.17 ± 0.48 mL/min (Group II), 1.07 ± 0.52 mL/min (Group III) proven significant statistically ( *p* = 0.001).	There is a relationship between nutrition status and salivary flow.	Fair
6	Hashem et al (2016) 23	Effect Of childhood malnutrition on salivary flow and pH	Cross-sectional	400 (3–12 y)	Salivary flow pH	Underweight and stunting decrease the flow of stimulated saliva secretion ( *p* = 0.252; *p* = 0.000) and does not affect the unstimulated salivary flow and saliva pH with a nonsignificant result statistically.	There is a correlation between underweight or stunting to stimulated salivary flow.	Poor
7	Muhammad et al (2015) 26	Prevalence of dental caries, gingival status, and enamel defect and its relation to nutritional status among kindergarten children in Sulaymaniyah city	Cross-sectional	914 (4 and 5 y)	Dental plaque (Silness and Löe) Dental calculus (Green and Vermillion)	High plaque index is found in underweight children and stunted children compared with those with good nutrition and is proven significantly. Even though the calculus is low at this age, a significant difference is only present in children with wasting and children with good nutrition.	Malnourished children have higher plaque index and calculus compared with children with good nutrition. Nevertheless, calculus is only proven significant in children with wasting.	Fair
8	Angulo et al (2012) 29	Childhood stunting and caries increment in permanent teeth: a three-and-a-half-year longitudinal study in Peru	Cohort	121 (7–9 y)	OHI-S	There are more stunted children with poor oral hygiene than nonstunting children (96% in stunted children and 84% in nonstunting children), which is proven significant ( *p* = 0.008).	Stunted children have poor oral hygiene compared with nonstunting children.	Good
9	Radhi et al (2012) 27	Salivary vitamins and total proteins, concerning caries experience and gingival health, according to nutritional status of a group of five-year-old children	Cross-sectional	60 (5 y)	Dental plaque (Silness and Löe) Saliva content	Nutrition is measured by using the high indicator for age. High moderate on PI is found in malnourished children compared with those with good nutrition, with a highly significant difference statistically (PI: *t* = 7.340 df = 58, *p* < 0.0001). The amount of vitamin and total protein of saliva is significantly lower in malnourished children than children with good nutrition ( *p* < 0.001). Vitamin A, C, and E in malnourished children negatively correlate to PI in malnourished children, whereas to protein, it has a positive correlation *p* < 0.001.	Chronic malnutrition in childhood (stunting) is correlated with saliva hypofunction. Malnourished children have high plaque index and lower saliva composition (vitamin and protein). This can be a risk factor for dental caries and gingivitis.	Good
10	Hasan et al (2010) 25	The effect of nutritional status on dental caries in relation to salivary flow rate, pH, inorganic phosphorus, calcium, copper and lead among five years	Cross-sectional	163 (5 y)	Salivary flow pH Saliva content	The moderate score of salivary flow is far lower in malnourished children compared with those with good nutrition ( *p* < 0.01). The average pH score is significantly lower in malnourished children than children with good nutrition ( *p* < 0.01). All elements of inorganic saliva in this research (phosphorus, calcium, copper, lead) are lower in malnourished children compared with children with good nutrition with the significant difference in phosphorus ( *p* < 0.05), and highly significant in calcium, copper, and lead ( *p* < 0.01).	Malnutrition affects the decrease of salivary flow, pH, phosphorus, calcium, copper, and lead.	Poor
Abbreviations: ANOVA, analysis of variance; APR, adjusted prevalence ratio; CI, confidence interval; ECC, early childhood caries; OHI-S, Simplified Oral Hygiene Index; PI, dental plaque; SFR, salivary flow rate.								

### Description of the Study Characteristics

The total number of articles that have been selected and reviewed is ten articles with variable study designs and quality. No meta-analysis was performed because of the heterogeneity of the identified studies. The population of the studies varied from 60 to 914 children depending on the article. Moreover, the types of studies were different: eight studies used a cross-sectional study design, 20 21 22 23 24 25 26 27 28 and one cohort study. 29 There were also different types of the parameter used to analyze oral health. While some authors use Oral Hygiene Index-Simplified (OHI-S), 23 28 29 some others use dental plaque index (Silness and Löe) and or dental calculus index (Greene and Vermillion). 26 27 Dental caries, as the most common oral disease, was also a parameter for defining oral health mention by one author. 28 Salivary flow rate also seen used by authors to measure oral health. 21 22 30 31

Assessing the quality of evidence contained within a systematic review is as important as analyzing the data within. NIH quality assessment tool, as shown in Table 1 revealed that four articles were of good quality, 22 27 32 33 two other articles were in the fair category, 13 20 while four articles were of poor quality or had high potential for bias. 7 23 24 31

## Discussion

The correlation between growth stunting and oral health seems quite evident, and there is significant scientific evidence that points to this direction. In this study, oral health was defined by the finding of oral hygiene, oral disease (dental caries), salivary flow rate, and content. Oral hygiene was measured by using OHI-S by three authors. 24 33 34 The study by Angulo et al (2012) stated that poor oral hygiene was higher in stunted children (96%) than healthy children (84%). 33 Other studies revealed that in 25 children with growth stunting, 24 of them have poor oral hygiene as the remaining with a moderate level. 24 A different matter was stated by Folayan et al in 2019 that associated stunting with early childhood caries (ECC) and related oral hygiene. Stunting is one of the risk factors for ECC. Of all the samples, there were no children with poor oral hygiene. So this study links stunting with ECC and moderate oral hygiene. 34 Plaque and calculus examination by Muhammad et al (2015) in his study revealed that a high plaque index was found in children with growth stunting compared to the well-nourished group, and this is significant. Meanwhile, the calculus index in this study was still shallow and only proved significant for the wasted category. 26 A high mean plaque index was also found in children with growth stunting compared to well-nourished children in the study of Radhi et al (2012) with a statistically very significant difference. 27

Since Saliva plays a vital role in maintaining oral health and functions, 35 the condition of moderate to poor oral health in stunted children can be caused by the hypofunction of the salivary glands, which is associated with a lack of nutrition in children. Hypofunction in the salivary glands can cause a decrease in salivary flow rate, reduced buffer capacity, and decreased composition of the Saliva. 33 In good conditions, these salivary protective functions can help the clean surface of the teeth, one of which is from bacteria that accumulate in plaque food debris. 36 Singh et al, in a 2018 study about salivary flow rates, revealed a decrease in stimulated and unstimulated salivary flow rate in children with growth stunting compared to well-nourished children. 21 Other studies also showed the same thing because there was a negative correlation between salivary flow rate and nutritional status. This negative correlation means that a decrease in salivary flow occurs with a worsening child’s malnutrition. 22 A similar statement is also stated by Hasan et al, with his study shows a moderate value of salivary flow rate in children with growth stunting was much lower than that of well-nourished children. 31 Meanwhile, Hashem et al (2016) have different results from Singh et al. In their study, growth stunting reduces the stimulated salivary flow rate and has no effect on the unstimulated salivary flow rate. 23

In chronic malnutrition, which reflects growth stunting, the flow rate of saliva tends to decrease, leading to disruption of the protective function of Saliva in the oral cavity. This may reduce the ability of the oral cavity to withstand infection and the acid-buffering capacity of plaque that is directly related to caries. 22 Regarding the study results by Hashem et al, where the unstimulated salivary flow rate was found not to affect, this could be due to changes in the weight of the parotid glands and decreased density of β-adrenoceptors. The parotid glands account for 50% of stimulated saliva secretion and only 20% for unstimulated Saliva. 37 Also, Hashem’s study was of a poor quality allowing a high potential for bias.

The pH test of saliva by Hashem et al and Hasan et al showed different results. Hashem stated that the pH of saliva in children with growth stunting was not affected, while Hasan stated that the mean pH value was significantly lower in stunted children than in well-nourished children. 23 31 Saliva has an essential role in maintaining normal pH in the oral cavity and plaque. 38 The change in pH of dental plaque will occur after food is consumed; a decrease in pH below the critical value will lead to demineralization of the tooth surface, leading to caries formation. 39 However, there was a drawback to the two studies reviewed, both of which had poor journal quality or allowed a high potential for bias.

Apart from examining the pH of saliva, Hasan et al also examined the inorganic content of saliva, which was phosphorus, calcium, copper, and lead. 31 The calcium and phosphorus content of Saliva can diffuse in a tooth and increase tooth resistance to caries. Calcium content can also be part of the formation of plaque consistency and affects the mineralization and demineralization process. 38 In Hasan’s study, all inorganic salivary elements in this study (phosphorus, calcium, copper, and lead) were lower in the malnutrition group than in the fantastic nutrition group, if significant differences phosphorus and highly significant for calcium, copper, and lead. 31

Vitamins A, C, E, and total protein, such as other content of saliva, were investigated by Radhi et al. The results showed that the value of vitamins and total salivary protein was significantly lower in malnourished children than in well-nourished children. Vitamins A, C, and E in malnourished children negatively correlated with the plaque index, while protein positively correlates with the plaque index. 27 The antioxidant system of saliva plays a vital role in building resistance and caries’ susceptibility due to its free radical scavenging action. Also, the vitamins studied in this study affect the oxidative metabolism of carbohydrates in plaque to affect the metabolism of bacteria, which can lead to cell death. 27 Thus, a decrease in vitamins may allow reduced salivary functions. Protein in Saliva also has a multifunctional role because it can work for the host as well as against the host. In connection with the results of the presentation of Radhi’s research, which states that protein and plaque have a positive correlation, this can be due to the function of proteins that play a role in the adhesion process of microbes. This microbial adhesion process initiates the development of pathogenic plaques. 40

The results of the above studies are in line with the review conducted by Achmad et al. The conclusion retrieved from various previous articles was that there was a relationship between dental conditions and stunting in children. Malnourished children have atrophic salivary glands that reduce salivary secretion and interfere with saliva’s function to buffer and cleanse the oral cavity. 7

## Conclusion

Based on the description above, it can be concluded that there is a correlation between growth stunting and oral health in children. This can be seen through the oral hygiene categorizing as moderate-poor through the OHI-S examination and the high plaque index found in selected studies. Also, it was found that there was a decrease in salivary flow rate and the composition of saliva in children with growth stunting. Dental caries is the most common contributor to oral disease, which implies decreased oral health and is correlated in children with growth stunting.
